# Alterations in Cell Mechanics by Actin Cytoskeletal Changes Correlate with Strain-Specific Rubella Virus Phenotypes for Cell Migration and Induction of Apoptosis

**DOI:** 10.3390/cells7090136

**Published:** 2018-09-13

**Authors:** Martin Kräter, Jiranuwat Sapudom, Nicole Christin Bilz, Tilo Pompe, Jochen Guck, Claudia Claus

**Affiliations:** 1 Biotechnology Center, Center for Molecular and Cellular Bioengineering, Technische Universität Dresden, 01307 Dresden, Germany; martin.kraeter1@tu-dresden.de (M.K.); jochen.guck@tu-dresden.de (J.G.); 2 Institute of Biochemistry, Leipzig University, 04103 Leipzig, Germany; jiranuwat.sapudom@uni-leipzig.de (J.S.); tilo.pompe@uni-leipzig.de (T.P.); 3 Department of Dermatology, Venerology and Allergology, University Clinic of Leipzig, 04103 Leipzig, Germany; 4 Institute of Virology, University of Leipzig, 04103 Leipzig, Germany; Christin.Emmrich@medizin.uni-leipzig.de

**Keywords:** wound healing, gap closure assay, actin stress fibers, real-time deformability cytometry, cytochalasin D, single cell tracking, cell stiffness

## Abstract

The cellular cytoskeleton is central for key cellular functions, and as such is a marker for diseased and infected cell states. Here we analyzed infection with rubella virus (RV) strains with respect to phenotypes in cellular mechanical properties, cell movement, and viral cytopathogenicity. Real-time deformability cytometry (RT-DC), as a high-throughput platform for the assessment of cell mechanics, revealed a correlation of an increase in cortical filamentous-actin (F-actin) with a higher cellular stiffness. The additional reduction of stress fibers noted for only some RV strains as the most severe actin rearrangement lowered cell stiffness. Furthermore, a reduced collective and single cell migration speed in a wound healing assay was detected in addition to severe changes in cell morphology. The latter was followed by activation of caspase 3/7 as a sign for induction of apoptosis. Our study emphasizes RT-DC technology as a sensitive means to characterize viral cell populations and to implicate alterations of cell mechanical properties with cell functions. These interdependent events are not only promising options to elucidate viral spread and to understand viral pathologies within the infected host. They also contribute to any diseased cell state, as exemplified by RV as a representative agent for cytoskeletal alterations involved in a cytopathological outcome.

## 1. Introduction

Rubella virus (RV) is the only member of the genus *Rubivirus* of the *Togavirus* family. The single-stranded RNA genome in plus-strand orientation encodes two non-structural (P150 and P90) and three structural (the envelope glycoproteins E1 and E2 and the capsid protein) proteins. While postnatal infections are generally mild or asymptomatic, perinatal infections of susceptible women during the first trimester of pregnancy can result in severe malformations of the unborn child known as congenital rubella syndrome (CRS). Besides abnormalities in the heart, eye, and ear, vascular and endothelial pathologies have been noted [1,2,3,4]. Despite the availability of an effective vaccine, RV is still a cause of outbreaks [5,6] and CRS cases occur even in countries of the Western world [7]. Although these cases are mainly import-related, it emphasizes that RV as an efficient teratogen is of ongoing relevance. The mechanisms and signaling pathways that lead to the strictly human-specific pathophysiological mechanisms behind CRS are still unknown, but contributing factors were discussed [4,8]. Among those factors was the reduction and rearrangement of actin filaments in discrete clumps in viral protein-enriched areas [9]. Furthermore, filamentous actin (F-actin) stress fibers are disrupted at late time points of RV infection [10]. All viral proteins involved in RV replication, namely the replicase proteins P90 and P150, and the capsid protein [11], were found to co-localize with the actin cytoskeleton [10,12].

F-actin is a major contributor to cellular mechanics [13]. This is a physical property of a cell, which can be altered in response to cellular stress or changes in cell functions. Thus, it is hypothesized that cell functional alterations by RV infection can be assessed by the cell mechanical phenotype to define features of virus populations. The cell mechanical phenotype is an under-investigated aspect in virology, but its implication in multiple cellular functions is of high relevance for the characterization of the pathobiology of a number of virus infections, including RV. The mechanical state of a cell influences its capacity for an immunologic response in addition to its morphology and migratory capacity [14,15,16]. Furthermore, cell mechanics have previously been evaluated as a biophysical marker for pathological alterations. They refer to various aspects of human diseases, including cancer invasion, anti-cancer drug resistance [17], and changes in the morphology of blood cells as a consequence of hereditary or malignant diseases [18]. Cell mechanics were shown to be relevant in the identification of red blood cells infected by *Plasmodium falciparum* [18] and for the assessment of immune cell activation during Salmonella infection [19]. Furthermore, the assessment of cell mechanics identified changes in cellular viscoelasticity as a supportive process during infection with enterohaemorrhagic *Escherichia coli* (EHEC) [20].

RV is a promising representative agent for mechanical phenotyping of virus-infected cells by real-time deformability cytometry (RT-DC) [21]. Besides the above-mentioned findings regarding changes in F-actin structures, RV displays strain-specific differences in its replication kinetics and effects on its host cell, including metabolic alterations [22]. These differences could contribute to mechanisms of viral pathogenicity and reflect principles of virus–host coevolution. Mechanical phenotyping performed in this study emphasized this notion and revealed profound but differential effects of RV strains on cell mechanics. The significant alterations in cellular stiffness induced by RV were also evident by a reduction in collective and single cell migration and an elongated cell shape. The latter was positively correlated with induction of apoptosis.

This study emphasizes the potential of cell mechanics to assess viral population dynamics through large scale analysis of cell state transition events during natural virus infection. Moreover, it can serve as a new parameter to make predictions not only for the infected, but also the diseased cell state involving the cytoskeleton network and its associated membranes.

## 2. Material and Methods

### 2.1. Reagents

The monoclonal antibody Mab to E1 (Viral Antigens Incorporation, Memphis, TN, USA) was used for immunofluorescence analysis of viral E1 expression. Donkey IgG anti-mouse IgG (H + L)-Alexa Fluor Cy3 used as secondary antibody for immunofluorescence analysis was from Dianova (Hamburg, Germany).

### 2.2. RV Strains

Besides RV laboratory strain F-Therien (Therien) and vaccine strain RA27/3, various low-passaged clinical isolates were used, representing clade 1 (Rub 1B (1B), Rub 1C (1C), RVi/Gdansk.POL/14.07_07-00426 (07-00426, genotype 1E), RVi/Prahova region.ROU/25.03_03-03703 (03-03703, genotype 1G)) and clade 2 (RVi/Wuerzburg.DEU/47.11_12-00009 (Wb-12, genotype 2B) and RV 114 (2B)) genotypes.

### 2.3. Cell Culture and Virus Infection

The Vero (ATCC, CCL-81) cell line was cultivated in Dulbecco’s modified Eagle’s medium (DMEM; Gibco, Thermo Fisher Scientific, Waltham, MA, USA) with high glucose, supplemented with 10% fetal calf serum (FCS) and penicillin/streptomycin in a humidified atmosphere with 5% CO_2_. Human umbilical vein endothelial cells (HUVECs, pooled, PromoCell, Heidelberg, Germany) were cultured in endothelial cell basal medium 2. Cells were incubated in the respective cell culture medium for 2 h with RV strains at a multiplicity of infection (MOI) of 3 (Vero), unless otherwise stated. An MOI of 10 was applied to HUVEC cells. This was followed by incubation in fresh medium until further analysis. At 48 h post-infection (hpi), before the onset of CPE, Vero cells were processed for assessment of cytoskeleton rearrangements, RT-DC analysis, cellular mobility measurements, and wound healing capacity. For mRNA expression studies, MOI 5 was applied and samples were collected at 72 hpi to maximize any possible RV-associated effects. Virus titration was performed by plaque (RV strains Wb-12, RA27/3, Therien, and Rub 1B) or focus-forming assay (all remaining RV strains) as published [23].

### 2.4. Relative Quantification of Cellular mRNA

Mock- and RV-infected Vero cells grown in 35-mm dishes were subjected to extraction of total RNA through Trizol reagent (Thermo Fisher Scientific). For RNA purification, the Direct-zol^TM^ RNA kit (Zymo Research, Irvine, CA, USA) was used, which included column-based digestion of cellular DNA by DNase I according to manufacturer’s instructions. RNA quality was evaluated on a fragment analyzer (Advanced Analytical, Ankeny, IA, USA). The respective cDNA was obtained through reverse transcription of total RNA (1.5 to 2 µg) with Oligo(dT)_18_ primer and AMV reverse transcriptase (Promega) at 42 °C for 1 h, which was followed by an incubation step at 70 °C for 10 min. Quantitative real-time PCR (qRT-PCR) was carried out in a carousel-based LightCycler 2.0 (Roche, Basel, Switzerland) based on GoTaq qPCR Master Mix (Promega, Mannheim, Germany) with a 1:5 dilution of the cDNA samples and 1 µg bovine serum albumin (BSA). Caspase 3 mRNA expression level was determined by the 2^−ΔΔ*CT*^ method after amplification of cDNA samples with published oligonucleotides 5′-AATTGTGGAATTGATGCGTGATG-3′ (forward) and 5′-CTACAACGATCCCC TCTGAAAAA-3′ (reverse) [24], at an annealing temperature of 54 °C. mRNA levels were normalized to hypoxanthine guanine phosphoribosyl transferase 1 (HPRT1) [25].

### 2.5. Immunofluorescence Analysis and Staining of the Actin Cytoskeleton

Cells cultivated on glass slides were washed with phosphate buffered saline (PBS) prior to fixation with 2% (*w*/*v*) paraformaldehyde (PFA) in PBS for 15 min. For visualization of dead cells in the RV-infected monolayer, the fixable Zombie aqua dye (Biolegend, San Diego, CA, USA) was added prior to fixation according to manufacturer’s instructions. Cells were washed with PBS and permeabilized for 5 min with ice-cold methanol thereafter. This was followed by blocking with 1% BSA in PBS (*w*/*v*) for 30 min at 37 °C in a humidified chamber. Incubation with primary anti-E1 antibody at a 1:200 dilution was performed for 60 min at 37 °C. After three washes with PBS, samples were incubated with the secondary antibody at a 1:200 dilution for 30 min. Plates were washed three times with PBS thereafter. A 1:40 dilution of Alexa Fluor 488 phalloidin (Thermo Fisher Scientific) was added to label F-actin. As a DNA counterstain, Hoechst bisbenzimide 33258 (5 µg/mL, Thermo Fisher Scientific) was applied. Samples were mounted in Fluoromount G (SouthernBiotech, Birmingham, AL, USA). Images were taken using a Leica SP5 laser scanning microscope and processed using Corel DRAW x7 with slight alterations to brightness and contrast. For E1 protein localization we qualitatively analyzed the images taken for all RV strains.

### 2.6. Activation of Caspase 3 and 7

At 48 hpi mock- and RV-infected Vero cells were plated in 24-well plates. Twelve hours after plating, activation of caspase 3/7 was assessed through the CellEvent caspase-3/7 Ready Probes^®^ reagent (Thermo Fisher Scientific) according to manufacturer’s instructions. Upon activation caspase 3/7 cleave the non-fluorescent DEVD substrate and generate fluorescent signals, which were monitored using AxioObserver.Z1 microscope (Carl Zeiss Microscopy GmbH, Jena, Germany) equipped with incubation chamber and motorized in a fluorescence and phase contrast mode over 24 h at 10-min intervals. Images were 1388 × 1040 pixels in resolution with a x- and y-voxel size of 0.87 µm and were analyzed regarding cell aspect ratio with and fluorescent intensity at single cell level by home-built automated single cell tracking software [26]. Fluorescence signal of the DEVD substrate was analyzed from the whole detected cell area.

### 2.7. Real-Time Deformability Cytometry

RT-DC was performed as described previously [21,27]. Briefly, at 48 hpi mock- and RV-infected Vero cells were detached by trypsinization, washed with PBS, and suspended in PBS containing methylcellulose (MC-PBS, 0.5% (*w*/*v*)) at a concentration of 1–2 × 10^6^ cells/mL. Suspension was drawn in a syringe and connected to a microfluidic chip made from polydimethylsiloxane (PDMS) on a glass slide. The chip consisted of a central channel separated by two reservoirs. A second syringe, filled with MC-PBS, was used to hydrodynamically focus the cells inside the constriction of the chip, which was mounted to an inverted microscope. A syringe pump flushed cells through the channel at a constant flow rate of 0.16 µL/s. A high-speed camera imaged the cells at the end of the constriction. Through the flow profile cells were deformed into a characteristic bullet like shape. In real-time cell cross-sectional area (size (µm^2^)) and deformation were calculated. It has to be emphasized that deformation and size are not independent parameters for RT-DC. This implies that for two cells of identical mechanical properties, the larger cell will always deform more. A numerical model combining Stokes fluid dynamics with linear elasticity allows to disentangle the relationship of size and deformation and to deduce material properties, namely young’s modulus [28]. Within the scatter plots the color code originates from a kernel density estimation and indicates the event density (high density = red, low density = blue). Statistical comparison of Young’s modulus, deformation, and size was carried out with a one-dimensional linear mixed model analysis. One fixed and one random effect was considered, in order to analyze the difference between subsets of cells and to consider the replicates variance, respectively. *P*-values were determined by a likelihood ratio test, comparing the full model with a model lacking the fixed effect term [29].

### 2.8. Wound Healing Assay

Wound healing assay was performed in 24-well plates with two well culture inserts (ibidi GmbH, Martinsried, Germany). Then, 2.1 × 10^4^ Vero cells were plated per well of the insert and after incubation for 24 h cells were infected with RV strains at an MOI of 3. At 48 hpi the insert was removed and three wash steps with PBS were applied. The sequential images were continuously recorded over a period of 30 h at 15-min intervals using 10× objective (Zeiss) in phase contrast mode using Axio Observer Z1 microscope (Zeiss, Jena, Germany) equipped with incubation chamber and motorized stage. The incubation chamber was adjusted to cell culture condition at 37 °C, 95% humidity and 5% CO_2_ content. Images were 1388 × 1040 pixels in resolution with a x- and y-voxel size of 0.87 µm and were analyzed by ImageJ (NIH, Bethesda, MD, USA) using particle analysis function.

### 2.9. Analysis of Single Cell Migration and Morphology

At 48 hpi mock- and RV-infected Vero cells were collected by trypsinization and 10,000 to 25,000 cells were plated per well of a 24-well plate. After 24 h, the sequential images were continuously recorded over a period of 30 h at 15-min intervals using 10× objective (Zeiss) in phase contrast mode using Axio Observer Z1 microscope (Zeiss, Jena, Germany) equipped with incubation chamber and motorized stage. The incubation chamber was adjusted to cell culture condition at 37 °C, 95% humidity, and 5% CO_2_ content. Images were 1388 × 1040 pixels in resolution with a x- and y-voxel size of 0.87 µm and were analyzed by custom-made single cell tracking toolbox [26]. Cell morphological parameters, average cell speed and Euclidean distance of single cell trajectory were calculated. Cell aspect ratio (cell length divided by width) was used to characterize cell elongation. Euclidean distance was defined at the linear shortest distance between start and end point of single cell trajectory.

### 2.10. Statistics

Data in the manuscript are displayed as means ± standard deviation. Statistical significance of normalized mRNA expression levels was analyzed by paired Student’s *t*-test, otherwise samples were subjected to one-way ANOVA followed by Tukey’s post hoc analysis using Graph Pad Prism software (GraphPad Software, Inc., La Jolla, CA, USA). Asterisks in diagrams reflect level of significance (* *p* < 0.05, ** *p* < 0.01, *** *p* < 0.001, **** *p* < 0.0001).

## 3. Results

**Figure 1:**
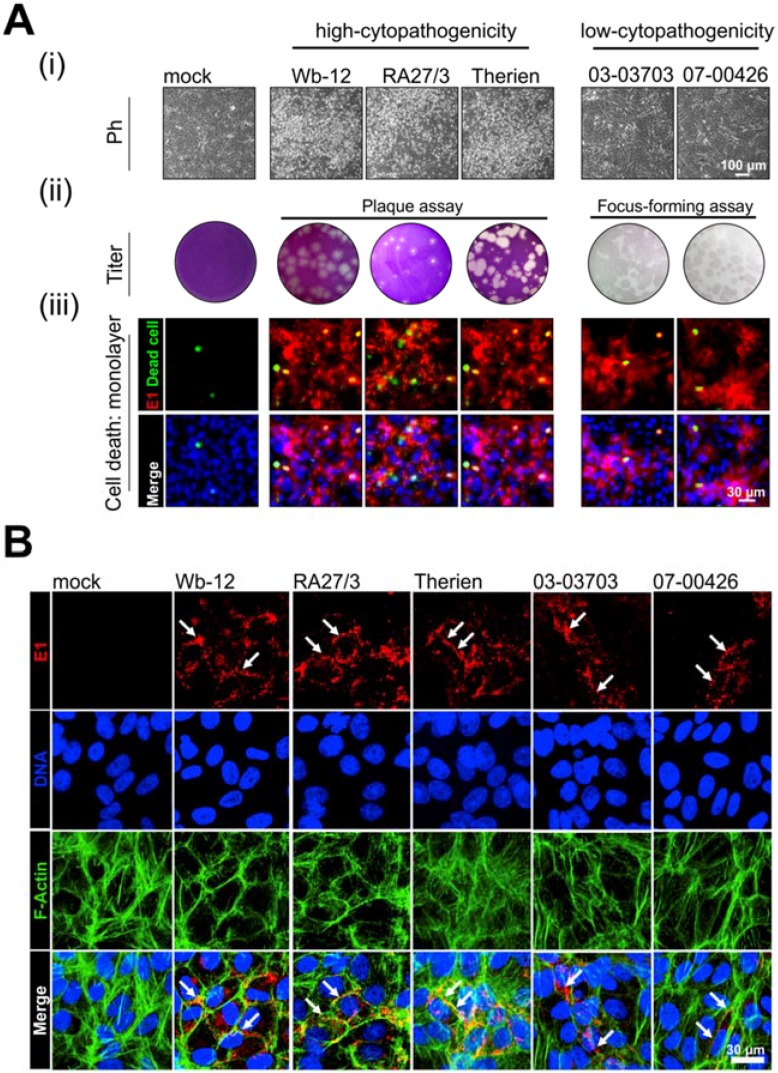
Rubella virus (RV) infection of Vero cells induces notable alterations in F-actin distribution. (**A**) Overview of the experimental background based on RV strains with a high (Wb-12, RA27/3, Therien) and low (03-03703 and 07-00426) level of cytopathogenicity. Cytopathogenicity after application of indicated RV strains at a multiplicity of infection (MOI) of 5 is visible through (i) a cytopathic effect (CPE) as indicated by cell rounding and detachment and (ii) determines whether viral titers are to be assessed by plaque or focus-forming assay, respectively. (iii) The number of E1-positive cells (shown in red) was determined by immunofluorescence analysis at 72 h post-infection (hpi). Some cells are overexposed to include cells with a lower level of E1 expression. Fixable Zombie aqua dye (shown in green) was applied to identify dead cells within the cell monolayer. Nuclei are depicted in blue. (**B**) Representative confocal maximum projections show viral E1 protein (red), F-actin (green), and nuclei (blue) distribution for mock- and RV-infected Vero cells (MOI of 3) at 48 hpi. Z-stacks of 2 µm height were acquired from culture substrate to cell center. Arrows highlight areas with altered distribution of cortical F-actin and E1 protein co-localization. Ph, phase contrast.

As an effective and robust cell model regarding RV infection, we used the epithelial cell line Vero. Due to the lack of a functional type I interferon system [30], effects related to its activation during RV infection could be excluded in the applied experimental setup. On susceptible cell lines such as Vero RV displays a cytopathogenic effect (CPE), but this is noted only after an incubation time of two to three days and at a strain-specific extent [31,32,33]. This is indicated in Figure 1A through the arrangement of RV strains into two groups, high-cytopathogenicity (Wb-12, RA27/3, and Therien strains) and low-cytopathogenicity (03-03703 and 07-00426 strains). CPE during RV infection is visible through cell rounding with subsequent detachment from the monolayer, which generates a population of cells floating in the supernatant (Figure 1A(i)). These morphological alterations are the basis for viral titer determination by plaque assay, whereas the low-cytopathogenicity group requires titer assessment by immunocolorimetric-based focus-forming assay (Figure 1A(ii)). Due to the cell detachment during RV infection, only a minor portion of dead cells can be detected in the infected cell monolayer (Figure 1A(iii)). Moreover, CPE development could contribute to an increase in the ratio of total number of particles to infectious particles, which was noted for the high-cytopathogenic strains Wb-12 and Therien, but not for the low-cytopathogenic strain 03-03703 [23].

As any disturbance with actin filament distribution could participate in CPE development, the CPE-susceptible cell line Vero is highly suitable to assess the consequences of the impact of high- and low-cytopathogenic RV strains on actin cytoskeleton. The RV strains analyzed in this study have been well characterized in a recent study with regard to replication rate, viral progeny generation, course of infection, and cytopathogenic potential [22]. Figure 1A(iii) is a visual summary of the data shown in this previous publication. As a reference to the already published data set, an MOI of 5 was applied and cells were assessed at 72 hpi. The amount of viral protein and the number of infected cells were reduced, albeit still sufficient, after infection with low-cytopathogenic as compared to high-cytopathogenic RV strains (Figure 1A(iii)), [22]. These well-characterized RV strains were chosen for this study due to their differences in CPE development [22], which could be indicative for differential effects on actin cytoskeleton.

### 3.1. Alteration in Actin Rearrangement Can Be Observed in Dependence on RV Strains

As the actin cytoskeleton influences cell functions and is involved in regulation of cell mechanics, we first analyzed its distribution pattern in RV-infected Vero cells. Cells were fixed and stained for F-actin and viral protein E1 at 48 h post-infection (hpi), before notable onset of CPE. As expected for epithelial cells grown on plastic surfaces, uninfected (mock) controls displayed dorsal stress fibers, transverse arcs and ventral stress fibers. In RV-infected cells as identified by viral E1 expression level, cortical actin bundles were found to be increased (Figure 1B). Furthermore, co-localization of the E1 protein with F-actin cortex was detected (Figure 1B). Especially in Wb12- and RA27/3-infected Vero cells a strengthened actin cortex ring was present while actin stress fibers were almost lost. This loss was less profound in 03-03703- and 07-00426-infected cells; however, cortical actin bundles were still increased in areas of E1 protein localization (Figure 1B). Therien-infected cells reflected a transitional state between the two groups with transversely connected thin actin bundles and cortical actin accumulation (Figure 1B).

In summary, the group one RV strains displayed the most profound alterations in actin cytoskeleton, which correlated with their higher level of CPE induction.

### 3.2. RV-Infected Cells Display an Altered Mechanical Phenotype

**Figure 2:**
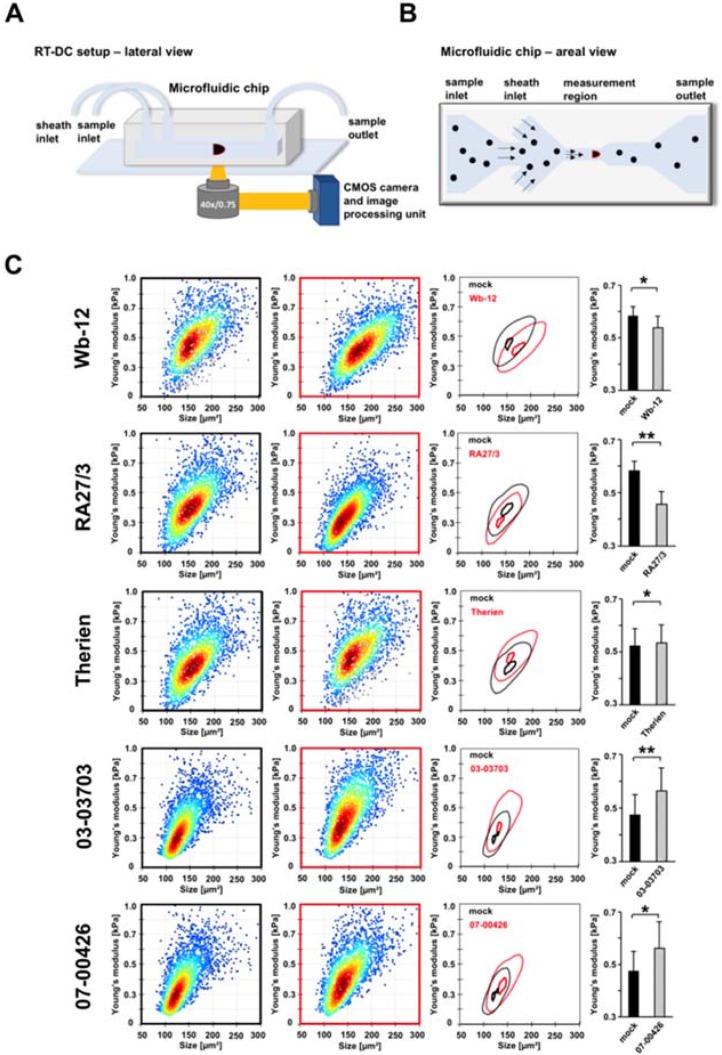
Mechanical characterization of RV-infected Vero cells by real-time deformability cytometry (RT-DC) identifies significant changes in cellular stiffness. (**A**) Experimental setup. Suspended cells were flushed through a microfluidic chip made from polydimethylsiloxane (PDMS) and imaged using a high-speed camera mounted through an inverted microscope. (**B**) Design of the microfluidic chip. The black dots represent suspended cells. Cell with a red contour represent deformation in the channel constriction within the region of interest. (**C**) Scatter plots show a representative measurement of mock (black border)- and RV (red border)-infected Vero cells. Corresponding contour plots highlight the mechanical and size differences of mock (black)- and RV (red)-infected cells. Here, the outer contour represents 50% event density and the inner contour 95%. Bar graphs are based on all experimental replicates (*n* = 3) performed for indicated RV strains and show statistical analyses of the cell stiffness (Young’s modulus) represented as mean ± standard deviation (SD). * *p* < 0.05; ** *p* < 0.01 vs. mock infected.

To test the hypothesis that RV infection of Vero cells leads to alterations in cell mechanical properties, cells were analyzed using RT-DC (Figure 2A) [21], and compared to uninfected mock controls. This high throughput technology allows a robust quantification of cell size and cell mechanical phenotype in suspension. Using a microfluidic chip cells were flushed through a narrow channel constriction and deformed through the flow-profile within the channel. This deformation is recorded by a high speed camera mounted to an inverted microscope (Figure 2A,B). Figure 2 shows representative scatter-plots of RV-infected cells compared to the corresponding mock controls (5000 cells measured at each condition). The results demonstrated that infection with all RV strains significantly altered cell mechanics. Moreover, the level of these alterations correlated with the extent of RV-induced changes of the actin cytoskeleton. The infection with RV strains Wb-12 and RA27/3 reduced cell stiffness (Figure 2), while cortical actin was increased and an almost complete loss of cytoplasmic actin filaments was noted (Figure 1). On the contrary, the retention of actin stress fibers in the presence of an increased cortical actin (Figure 1) was correlated with an elevated cellular stiffness under infection with 03-03703 and 07-00426 strain (Figure 2). An intermediate F-actin state, namely shown by thin transverse actin filaments and cortical bundling, was displayed by Therien infection, which correlated to a slight, but significant, increase in cell stiffness compared to mock controls. These findings indicate that the interplay between cortical actin and actin stress fibers plays an important role in defining the cell mechanical phenotype in RV infection. As control we also tested the association of disturbed F-actin assembly with Vero cell mechanics. We performed RT-DC measurements on cells after treatment with cytochalasin D at a concentration of 0.5 µM. As expected, the application of the actin polymerization inhibitor cytochalasin D resulted in a reduction in cell stiffness (Supplement Figure S1). Supplement Figure S2A collectively summarizes the mechanical properties of RV-infected Vero cells in comparison to the corresponding mock control in a representative fashion and emphasizes alterations in cell mechanics as a general characteristic of RV infection. To confirm these findings, HUVECs as primary human endothelial cells were exemplarily infected with strains Therien and 07-00426. HUVEC are only slightly susceptible to RV-associated CPE development [34]. Thus, they were used to assess whether changes in cell mechanics are cell line-specific and dependent on CPE development. As expected, we identified cell stiffening in both cases (Supplement Figure S2B). Interestingly, an increase in cell size was observed for infected HUVECs, which needs to be investigated in more detail.

Collectively our data suggest that RV infection of Vero cells significantly altered the cell mechanical phenotype, which occurred in correlation with the extent of RV strain-associated alterations of the actin cytoskeleton. Based on these findings we further followed the hypothesis that RV infection causes changes of important cellular functions related to the mechanical phenotype of cells, namely migration capacity and cell apoptosis.

### 3.3. RV-Infected Cells Possess a Reduced Collective and Single Cell Migration Capacity

**Figure 3:**
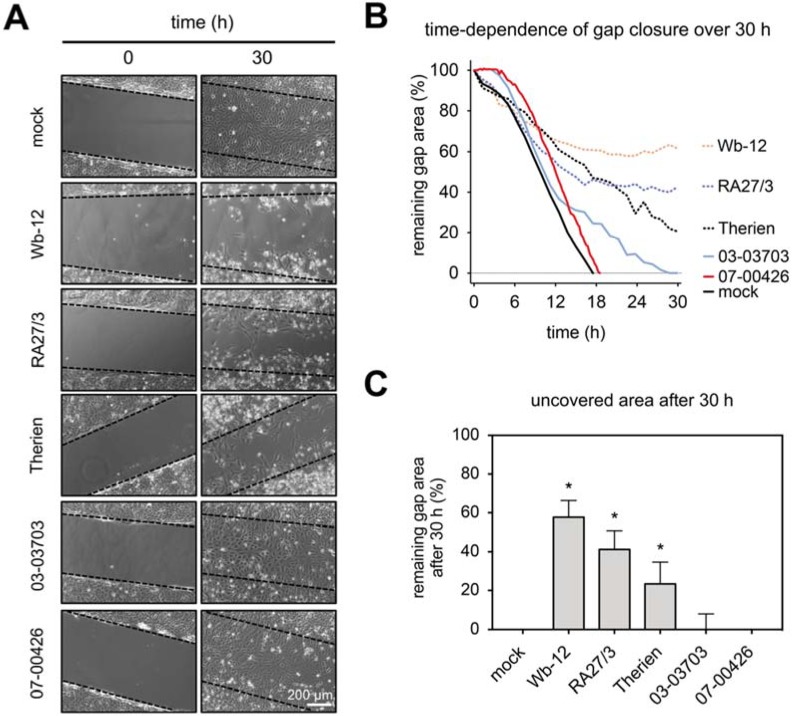
RV perturbs wound healing capacity of Vero cells. (**A**) Representative images of wound healing assay taken at 0 h and 30 h. Black dotted lines indicate the insert borders at the beginning of the assay and live cell images were recorded at 15-min intervals for 30 h. (**B**) Quantitative analysis of the time dependence of gap closure. The percentage of remaining gap area was calculated as the ratio of the remaining gap at the given time point and the original gap present at 0 h. The quantified values are presented as mean of three independent experiments (two positions per individual experiments). (**C**) Quantification of remaining gap area after 30 h. The quantified values were represented as mean ± SD of 3 independent experiments (two positions per individual experiment). * *p* < 0.05 vs. mock group.

For assessment of the impairment of cellular functions during RV infection, we first analyzed the collective migratory capacity of RV-infected Vero cells. We performed wound healing assays by the stopper insert method with a defined gap width of 500 µm. At 48 hpi the insert was removed and the time dependence of gap (wound) closure was monitored using phase microscopy at 15-min intervals over 30 h under standard cell culture conditions (Figure 3A). As shown in Figure 3B a completed gap closure was noted after 18 h for mock- and 07-00426-infected Vero cells. The infection with group one RV strains Wb-12 and RA27/3 induced loss of filamentous actin besides an increase in cortical actin and demonstrated a higher remaining gap area in the wound closure assay (Figure 3B,C). In contrast, cells infected with group two RV strains (03-03703- and 07-00426) showed only an increase in cortical actin and as such a complete gap closure with only a small delay. Therien-infected cells with an actin arrangement that was transitional between those two groups showed an intermediary gap closure rate in the wound healing assay. To demonstrate that actin filaments and their organization play a critical role in collective epithelial cell migration, we treated Vero cells with cytochalasin D at a concentration of 0.5 µM. No cell migration was observed after application of cytochalasin D to Vero cells and thus no gap closure was noted (Supplement Figure S3).

Data on collective cell migration demonstrated that especially the RV strains Wb-12, RA27/3, and Therien as representatives for the high-cytopathogenic RV strains caused a significant delay in gap closure rate in the wound healing assay. Only a minor effect was observed for the low-cytopathogenic strains 03-03703 and 07-00426. This indicates a reduced cell migratory capacity. The extent of this delay was correlated with the rearrangement of the actin cytoskeleton found in RV-infected cells.

**Figure 4:**
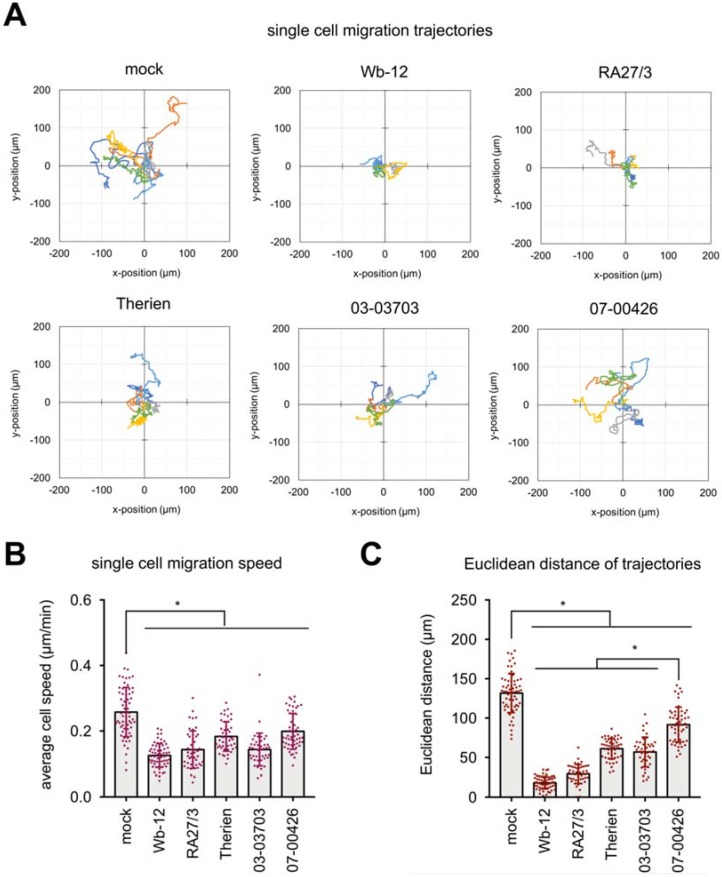
RV infection reduces epithelial cell migration as analyzed at the single cell level. (**A**) Representative migration trajectories of single cells. Cell migration was recorded every 15 min for 30 h and 6 cell trajectories per experimental conditions are depicted. Quantitative analysis of (**B**) single cell migration speed and (**C**) Euclidean distance of cell migration trajectories. Bars represent the means ± SD; *n* = 4 samples. * *p* < 0.05 vs. mock group. At least 60 cells per experimental condition were analyzed.

For further assessment of the reduction in migration capacity of RV-infected Vero cells, single cell migration assays were performed. Mock- and RV-infected Vero cells were re-plated on cell tissue culture plate at 48 hpi. After an incubation of 12 h live cell imaging was performed at 15-min intervals for 30 h under standard cell culture conditions. Single cell migration was automatically analyzed using a custom-made image analysis toolbox [26]. Representative trajectories of single cells shown in Figure 4A indicate that all RV strains triggered a significantly reduced migratory speed and Euclidean distance (straight distance between start and end point of cell trajectory). Under RV infection, the average migration speed was below 0.2 µm/min as compared to uninfected cells (average migration speed of 0.26 µm/min), (Figure 4B). While the reduction in single cell migration speed of RV-infected Vero cells was comparable among RV strains, analysis of Euclidean distance (Figure 4C) revealed a stronger reduction for cells infected with group one strains (Wb-12 and RA27/3) compared to mock or group two infection. Although cells infected with 07-00426 strain did not show any changes in collective cell migration in the wound healing assay, cell speed and Euclidean distance of single cell migration were significantly reduced compared to mock-infected cells. To demonstrate the importance of actin filaments and their organization for single cell migration, we treated Vero cells with cytochalasin D at a concentration of 0.5 µM. As shown in Supplement Figure S4, cells treated with cytochalasin D exhibited a strong impairment of cell migration, including cell migration trajectories (Figure S4B) and speed (Figure S4C).

To extend these observations and to assess whether noted effects are RV genotype specific, three additional low-passaged clinical isolates of RV were employed in single cell migration assays. Infection of Vero with Rub1B as a RV strain positive for CPE development resulted in a stronger reduction in single cell migration than infection with CPE-negative strains Rub 1C and RV 114 (Supplement Figure S5). This indicates that although CPE induction is present at a lower proportion among RV strains, the tremendous reduction in migratory speed as observed for Wb-12 strain was not due to a single evolutionary event during natural infection. It appears to represent a general property of RV strains. Additionally, Rub 1B belongs to genotype 1B, while Wb-12 is a genotype 2B virus, thus RV induced alterations of cell migration capacity were not restricted to a distinct genotype. Moreover, this analysis revealed no specific characteristics attributable to RV adaptation to Vero cells (Therien laboratory strain) or during attenuation (RA27/3 vaccine strain).

In summary, we could correlate the effect of RV infection on alterations of actin cytoskeleton and cell mechanical properties with collective and single cell migration data. All RV strains increased cortical actin and reduced single cell migration speed and Euclidean distance of cell migration trajectories. A loss of actin stress fibers was only noted for the group one RV strains which were positive for CPE development. This loss appeared to be associated with a lower level of cell stiffness and a significant impairment of cell migration. RV strains of group two exhibited a sole increase of cortical actin in connection with a higher level of cellular stiffness leading to a lower reduction in cell migration speed and Euclidean distance compared to group one.

### 3.4. Changes in Cell Morphology of RV-Infected Cells Are Associated with a Higher Apoptosis Rate

In single cell migration studies of RV-infected Vero cells, we observed a population with elongated cell morphology. These cells subsequently detached from the cell culture substrate, which could be due to either loss of cell-substrate contact or induction of apoptosis. Although RV is considered a low cytopathogenic virus, it can induce apoptosis on susceptible cell lines such as Vero and thus strongly impact cell function [31,35]. Induction of apoptosis in turn is correlated with morphological alterations and involves changes in actin distribution and stability [36].

**Figure 5:**
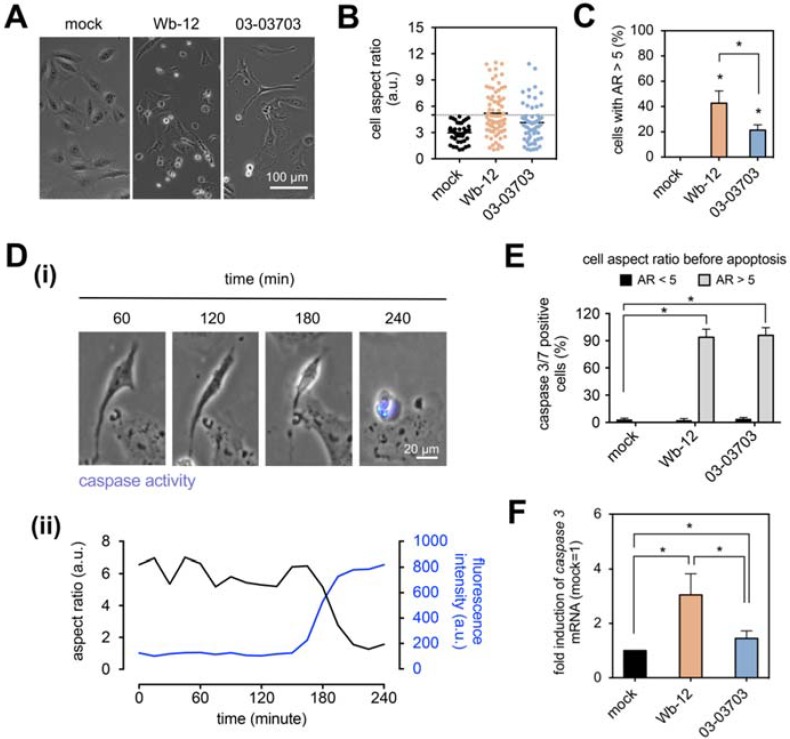
An elongated shape enriched in RV-infected Vero cells was associated with activation of caspase 3/7. At 48 hpi, Vero cells infected with indicated RV strains were freshly plated and analysis was started after 12 h of incubation. (**A**) Representative images of cell morphology were further analyzed for (**B**) cell aspect ratio and (**C**) the percentage of cells with an elongated phenotype (cell aspect ratio > 5) among Wb-12- and 03-03703-infected Vero cells. Custom-made single cell tracking software was applied. (**D**) Vero cells infected with Wb-12 were monitored for 24 h by fluorescence microscopy for activation of caspase 3/7 through cleavage of the DEVD peptide and subsequent generation of a fluorescence signal. Quantitative analysis was performed using custom-made single cell tracking software to correlate changes in cell aspect ratio with DEVD peptide cleavage. Illustrated as a function of time, (**D**(i)) representative images of caspase 3/7 activity and (**D**(ii)) a representative plot of cell aspect ratio (black line)/DEVD peptide cleavage (fluorescence signal; blue line) are highlighted. (**E**) Quantitative analysis of caspase 3/7 positive cells as a function of cell aspect ratio prior to induction of apoptosis. (**F**) Quantitative real-time PCR (qRT-PCR) analysis was applied to assess relative caspase 3 transcript levels at 72 hpi in Wb-12- and 03-03703-infected Vero cells. Hypoxanthine guanine phosphoribosyl transferase (HPRT1) was used for normalization. *n* = 4 samples. * *p* < 0.05 vs. mock group. At least 60 cells per experimental conditions for subfigures B, C, and D were analyzed.

To investigate whether RV infection leads to such morphological changes and whether this correlates with apoptosis induction, we studied the two RV strains Wb-12 and 03-03703 as representatives for group one and two. As noted before, both groups induced an increase in cortical actin in infected Vero cells, but only members of group one were associated with CPE development and loss of actin stress fibers. Figure 5A exemplarily shows the cell morphology after re-plating of Wb-12- and 03-03703-infected Vero cells. They revealed notable changes in their morphology, namely an elongated shape, which was quantified by cell aspect ratio (cell length divided by cell width). As shown in Figure 5B, a higher proportion of Vero cells with elongated morphology was present after infection with Wb-12 compared to 03-03703. To quantify the overall percentage of cells with an elongated morphology, we set the cut-off at an aspect ratio of 5, as uninfected cells exhibited a cell aspect ratio below 5 (Figure 5B). By doing this, we found that Vero cells infected with Wb-12 and 03-03703 significantly displayed elongated cell morphology of 44% ± 9% and 21% ± 4%, respectively (Figure 5C). Treatment of Vero cells with cytochalasin D at a concentration of 0.5 µM was used as a control to connect changes in actin cytoskeleton with the elongated cell morphology observed for RV-infected cells. After 4 h of incubation in the presence of the inhibitor cells were re-plated and observed under a phase contrast microscope after a further incubation period of 4 h. Comparable to RV infection, an elongated cell morphology was observed (Supplement Figure S6) emphasizing that rearrangements in actin cytoskeleton can lead to severe morphological changes.

Thereafter it was analyzed whether the altered cell morphology was correlated to apoptosis induction. For this approach, the non-fluorescent DEVD peptide was used. Its cleavage after activation of caspase 3/7 generates a fluorescence signal (Figure 5D(i)). Apoptosis induction was determined as a function of time by fluorescence microscopy at 15-min intervals for 24 h. We found that elongated cell morphology was associated with activation of caspase 3/7 (Figure 5D(ii)). Further, we quantitatively analyzed the percentage of caspase 3/7 positive cells in relation to cell aspect ratio. We found that the percentage of caspase3/7-positive cells within the cell population with an aspect ratio smaller than 5 (AR < 5) demonstrated no significant changes within mock- and RV-infected Vero cells. However, 95% of cells with elongated cell morphology (AR > 5) exhibited caspase 3/7 activation (Figure 5E). These results suggest that elongated cell morphology was associated with induction of apoptosis (as measured by caspase 3/7 activation). Among Wb-12-infected Vero cells the proportion of cells with elongated morphology was significantly higher than for the infection with 03-03703 strain (*p* = 0.0122, Figure 5C). Accordingly, a higher relative caspase 3 mRNA expression level was detected after infection with Wb-12 compared to 03-03703 through qRT-PCR analysis at 72 hpi (Figure 5F).

In conclusion, the presence of an elongated cell morphology in RV-infected Vero cell populations was positively correlated with activation of caspase 3/7. Similar morphological alterations were detected after treatment with cytochalasin D, which suggests that the elongated cell morphology was related to RV-induced changes in actin cytoskeleton and reflects the impairment of cellular functions by RV.

## 4. Discussion

Our study identified two phenotypes among RV-infected cells. One was present among RV strains positive for CPE induction (exemplified by Wb-12) with an increase in cortical actin, but a reduction in cytoplasmic actin filaments. These alterations were accompanied by a decrease in cellular stiffness and migratory capacity. After re-plating of these infected cells, a notable proportion with an elongated morphology and a subsequent induction of apoptosis was identified. The second phenotype applies to RV strains (exemplified by 03-03703) with a low level of CPE induction. In this group, alterations similar to group one were noted regarding cortical F-actin distribution, cell migration and cell morphology, but at a reduced level. During infection with the low-cytopathogenic strains cytoplasmic actin filaments were maintained, which was associated with an increase in cellular stiffness. RT-DC measurements were able to differentiate between these subtle differences among RV strains as measurements were undertaken in suspension, after detachment of cells. Thus, cell mechanics were assessed through changes in cell deformability that were based on rearrangements of cytoskeletal filaments.

The comprehensive analysis of the mechanical phenotype of RV-infected Vero cells has revealed a close association to RV infection-induced changes of the actin cytoskeleton. As cell functions such as cell migration or apoptosis induction are closely linked to cytoskeletal alterations, mechanical phenotyping might be sufficient to predict cell functional changes [37,38,39]. Thus, the use of high-throughput and label-free techniques for determination of cell mechanics will broaden our knowledge of cellular processes and their subversion by pathogens, e.g., it was already shown that a reduction in cell migration together with an increase in cell stiffness can occur as a cellular defense mechanism [19]. To our knowledge, our study is the first to contribute to the understanding of cell mechanics at a single cell level in the context of virus infection using RT-DC [21].

As the actin cytoskeleton is a major regulator of cellular stiffness, alterations in its structure influence the cellular mechanical phenotype, which in turn can be linked to distinct cellular function, thus being a potential diagnostic marker [40]. RV with its low level of cytopathogenicity allowed for the analysis of the impact of virus infection on changes of the actin cytoskeleton in the absence of cell lysis or extensive cell destruction. All RV strains examined in this study induced notable alterations in the distribution of the actin cytoskeleton. However, only a subset of RV strains reduced cellular stress fibers almost completely, which was correlated to a lower cellular stiffness compared to the respective mock control. The envelope glycoprotein E1 appeared to be localized in close proximity to cortical actin bundles, which were concentrated in distinct spots at the plasma membrane. This is in agreement with published data on disaggregation of actin filaments into foci during RV infection [9]. Here we have identified this as a common characteristic of different RV strains.

Despite the notable alterations in actin cytoskeleton pattern, the RV progeny generation was not affected by treatment with cytochalasin D as a potent inhibitor of actin polymerization [41]. However, and in contrast to its replication in cell culture, the rearrangement of the actin cytoskeleton could be required during infection of its human host to support virus spread. It was shown for endothelial cells that angiogenesis stimuli induced cytoskeletal changes and subsequently decreased cell stiffness in RT-DC measurements. This characteristic was correlated with a reduced cell–cell and cell–matrix adhesion as well as an increased leukocyte tissue infiltration and reduced barrier function in vivo [42]. As RV causes systemic infection and can be detected in the blood [43], a reduced endothelial cell barrier function could support viral spread to adjacent tissues. Future studies will help to understand the link of cell mechanics to viral spread within 3D cellular environments, including whole tissues.

Asides from oncogenic viruses in the field of tumorigenesis, only a few studies have addressed virus-induced alterations of the migratory capacity of cells. In addition to dsRNA as a component of RNA virus replication complexes (as discussed for dengue virus) [44], a virus particle itself (shown for herpes simplex virus) can alter migration potential of a cell [45]. The Hazara virus as an experimental model for Crimean–Congo hemorrhagic fever virus was analyzed on Caco-2 cells in an epithelial wound healing assay [46]. Comparable to our data on RV infection of epithelial Vero cells, Hazara virus inhibited migration rate and reduced expression of F-actin and several cytoskeleton-associated proteins [46]. In line with the slight variations identified for RV strains in the wound healing assay, published data on HCMV strains also indicate strain specific differences in virus induced alterations of cellular migratory capacity. The clinical strains VR1814 and TB40E decreased wound healing capacity of primary endothelial cells (ECs) [47]. Contrary, data on infection of microvascular endothelial cells with HCMV strains TB40/E-UL32 and Towne show enhanced collective cell migration [48,49]. This supports the growing awareness of the importance of different low-passaged virus isolates for virus infection studies as performed in this study. The infection of embryonic mouse brain slice cultures with Zika virus strains revealed that despite a difference of less than 10 amino acids within these strains variable infection patterns emerged [50].

We have correlated the impact of RV on actin cytoskeleton with cell mechanics and related this to important cell functions, namely cell migration and apoptosis. Thus, cell mechanics were successfully introduced as a novel means to characterize virus induced cellular alterations on a single cell level. Furthermore, this could be a valuable tool to identify sequences within the RV genome that direct phenotypic differences such as CPE development and changes in cell stiffness. Phenotypic properties such as CPE development and alteration of cellular metabolism are not associated with a distinct genotype [22]. Thus, they appear to be properties arising within different genotypes during natural infection. Cell mechanics and cell migration are also crucial for developmental processes [39]. The alterations in cell migratory capacity as identified in this study can contribute to malformations of the cardiovascular system that are noted for CRS, including patent ductus arteriosus [1,2,51]. Closure of the ductus arteriosus requires migration of undifferentiated smooth muscle cells [52]. The analysis of cell mechanics in the context of RV-associated alterations of developmental processes will be possible in future studies through the availability of iPSC-based model systems [53]. Our mechanical and functional studies emphasize that the parameters investigated in this study are required to fully elucidate RV-induced pathogenic alterations during both perinatal and postnatal infections. Moreover, they will broaden our current understanding of cellular alterations that contribute to efficient viral spread within its host.

## Supplementary Materials

Supplemenatary materials are available online.
