# Supplementary material for: Alterations in Cell Mechanics by Actin Cytoskeletal Changes Correlate with Strain-Specific Rubella Virus Phenotypes for Cell Migration and Induction of Apoptosis
Source: Cells. 2018 Sep 13;7(9):136. doi: 10.3390/cells7090136 (PMC6162683; doi:10.3390/cells7090136)
Supplement: Supplementary file 1 [file cells-07-00136-s001.pdf]

Supplementary information

Alterations in Cell Mechanics by Actin Cytoskeletal Changes Correlate with Strain-Specific Rubella Virus Phenotypes For Cell Migration and Induction of Apoptosis

Martin Kräter[1$], Jiranuwat Sapudom[2,3$], Nicole Christin Bilz[4], Tilo Pompe[2], Jochen Guck[1], Claudia Claus[4]

[$] Equally contributed

[1]Biotechnology Center, Center for Molecular and Cellular Bioengineering, Technische Universität Dresden, Dresden, Germany

[2]Institute of Biochemistry, Leipzig University, Leipzig, Germany

[3]Department of Dermatology, Venerology and Allergology, University Clinic of Leipzig, Leipzig, Germany

[4]Institute of Virology, University of Leipzig, Leipzig, Germany

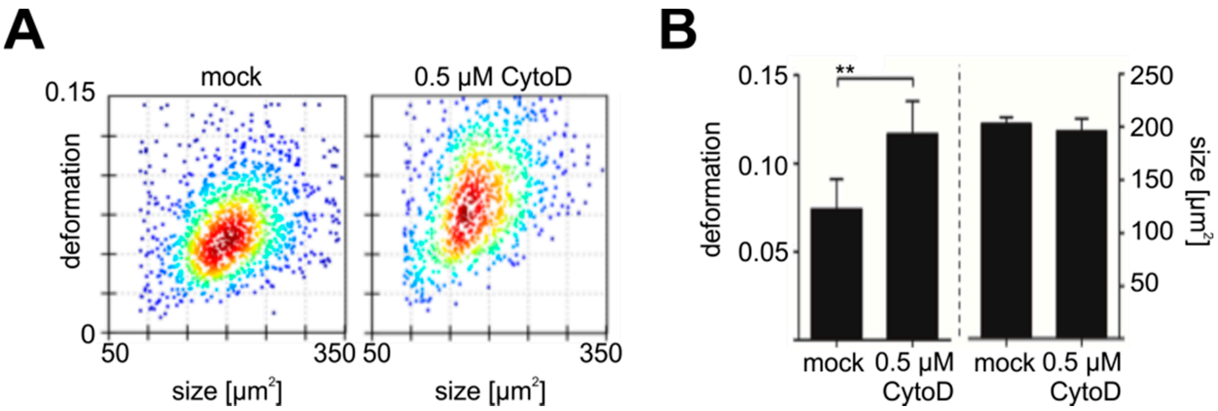

Supplement Figure S1: Cytochalasin D treatment of Vero cells reduced cell stiffness. Vero cells were incubated for 4 hours with 0.5 µM Cytochalasin D, detached by trypsinization and measured using RT-DC. **(A)** Representative scatter plots of cell deformation and size of mock control (solvent incubated) and Cytochalasin D (CytoD)-treated Vero cells. Cytochalasin D-treated Vero cells showed higher deformation in RT-DC, whereas cell size was not affected. This indicates treated cells to be softer. **(B)** Bar graphs show statistical significance of cell stiffness (deformation) and cell size represented as mean ± SD of 3 independent experiments. **p<0.01 vs. mock (solvent control).

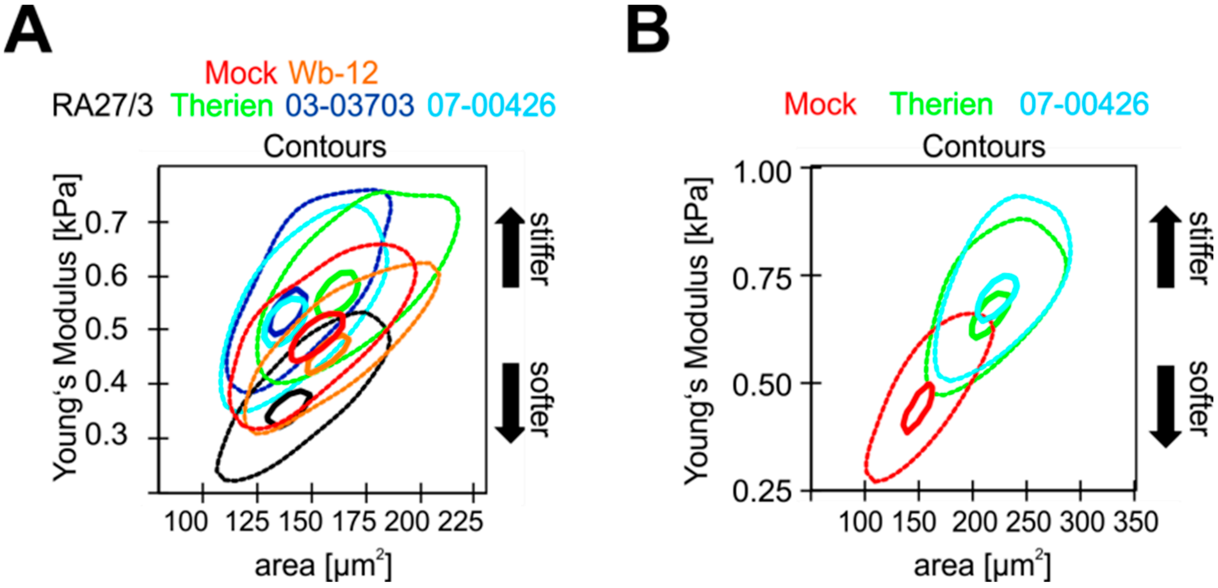

Supplement Figure S2: Representative contour plots of RV-infected (A) Vero and (B) HUVEC cells. Contour plots summarize the mechanical and size differences of mock- (shown in red) and RV-infected cells (colors indicate different RV strains). Here, the outer contour represents 50% event density and the inner contour 95%.

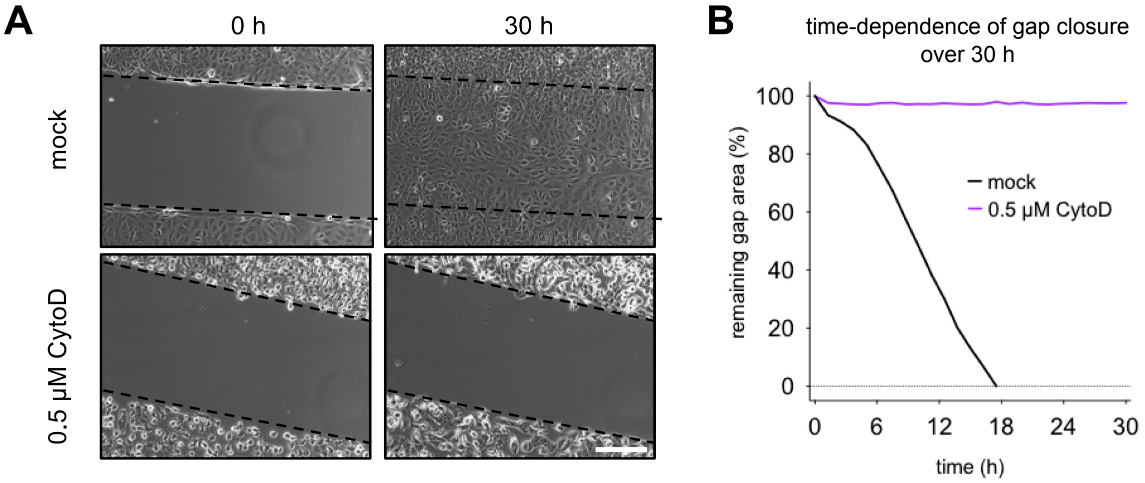

Supplement Figure S3. Effect of Cytochalasin D on collective cell migration (A) At 0 h and 30 h representative images of wound healing assay of mock- and Cytochalasin D-treated (CytoD, at 0.5 µM) cells were taken. Black dotted lines indicate the insert borders at the beginning of the assay and live cell images were recorded at 15-min intervals for 30 h. **(B)** Quantitative analysis of the kinetic of gap closure. The percentage of remaining gap area was calculated as the ratio of the remaining gap at the given time point and the original gap present at 0 h. The quantified values are presented as mean of 3 independent experiments (2 positions per individual experiments). *p<0.05 vs. mock (solvent control).

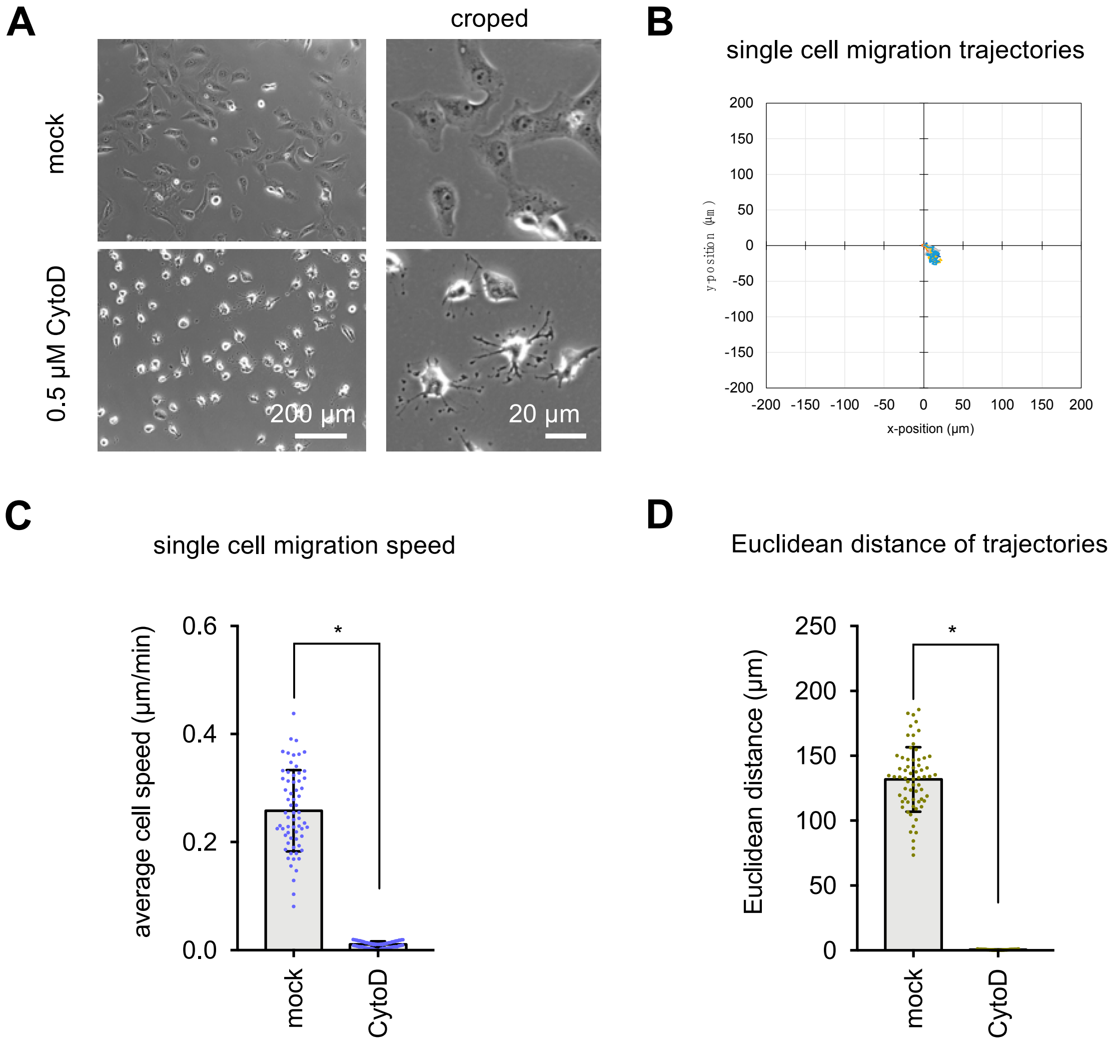

Supplement Figure S4. Effect of Cytochalasin D on single cell migration (A) Representative image of cell morphology with and without treatment with 0.5 µM Cytochalasin D (CytoD). For overall cell distribution a lower-magnification image is given, which is complemented by a cropped (enlarged) image for better visualization **(B)** Representative migration trajectories of single cells. Cell migration was recorded every 15 minutes for 30 h and 6 cell trajectories per experimental conditions are depicted. Quantitative analysis of **(C)** single cell migration speed and **(D)** Euclidean distance of cell migration trajectories. Bars represent the means ± SD; n=4 samples. *p<0.05 vs. mock (solvent control). At least 60 cells per experimental condition were analyzed.

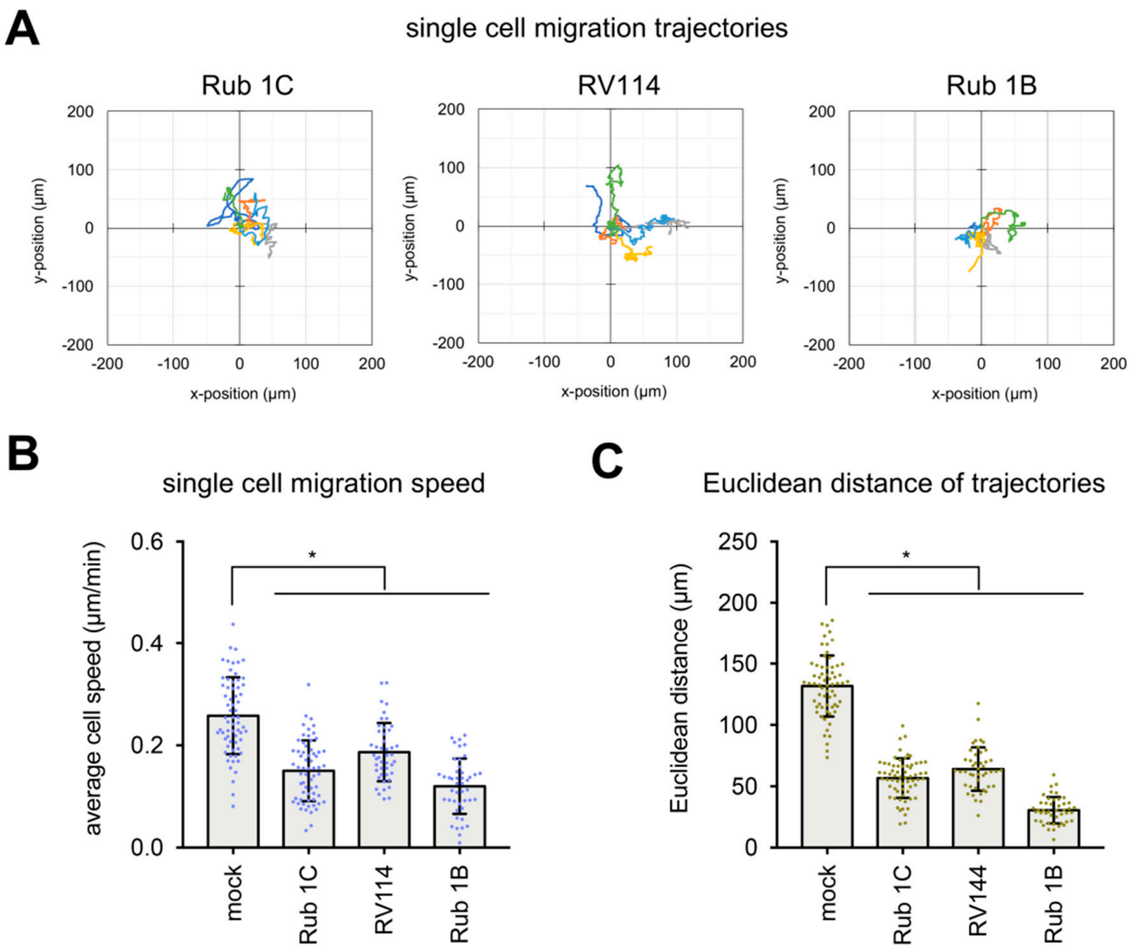

Supplement Figure S5: Effect of RV infection on epithelial migration at single cell level. (A) Representative cell migration trajectories of single cells. Cell migration was recorded every 15 minutes for 30 h and 6 cell trajectories per experimental conditions are depicted. Quantitative analysis of **(B)** single cell migration speed and **(C)** Euclidean distance of cell migration trajectories. Bars represent the means ± SD; n=4 samples. $^{*}p<0.05$ vs. mock group. At least 60 cells per experimental condition were analyzed.

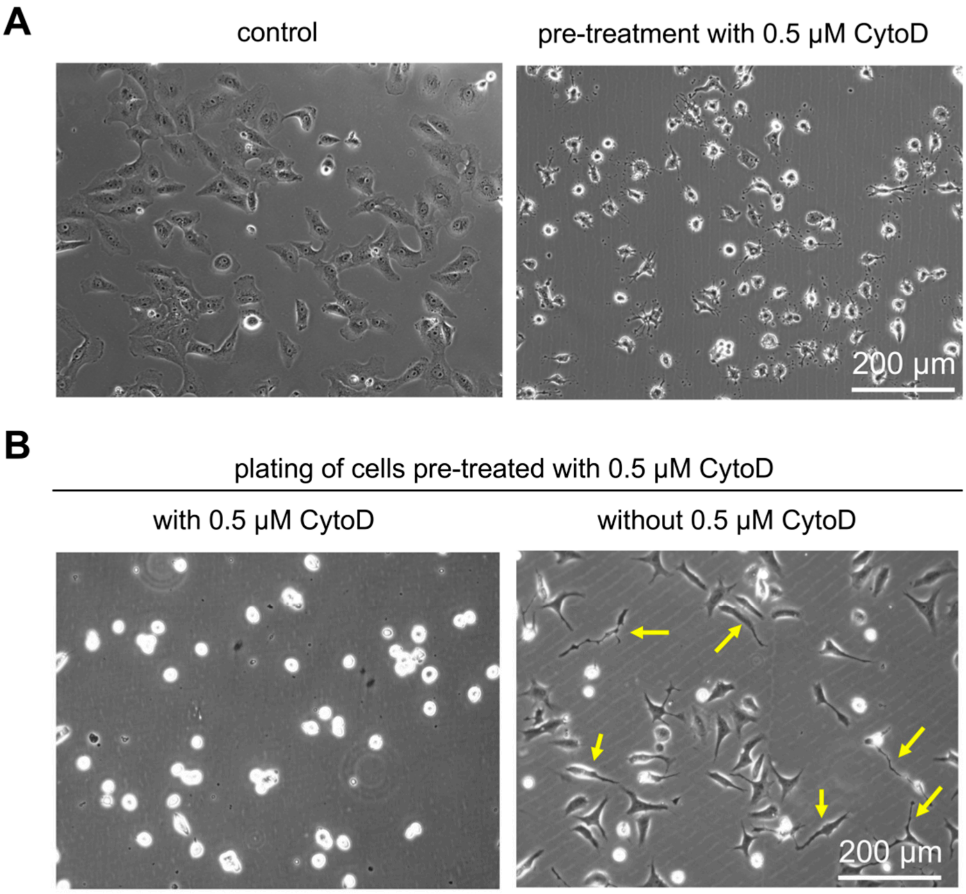

Supplement Figure S6: Treatment of Vero cells with Cytochalasin D prior to cell plating results in elongated cell morphology. Representative images of cell morphology **(A)** without and with pre-treatment with 0.5 μM Cytochalasin D (CytoD) for 2 h and **(B)** after plating of pre-treated cells in the further presence and absence of 0.5 μM Cytochalasin D (CytoD). Yellow arrows show the elongated cell shape.
